# The Important Role of CoQ_10_ in Aging

**DOI:** 10.3390/antiox8120570

**Published:** 2019-11-20

**Authors:** Guillermo López-Lluch

**Affiliations:** Centro Andaluz de Biología del Desarrollo, Universidad Pablo de Olavide-CSIC-JA, and CIBERER, Instituto de Salud Carlos III, 41013 Sevilla, Spain; glopllu@upo.es; Tel.: +34-954-9384

Coenzyme Q_10_ (CoQ_10_) is an essential lipid present in all cell membranes. This molecule is formed by a polar head bound to a ten units isoprene tail. Its dual role as key electron transfer factor in the mitochondria—as the only lipidic member of the respiratory electron transport chain, and as the main antioxidant in the prevention of oxidative damage in cell membranes—makes it an essential molecule in life and survival. In fact, all the aerobic organisms show this molecule or derivatives with lower isoprene chain. Depletion of the levels of this molecule during aging can aggravate mitochondrial dysfunction accelerating the progression of aging or age-associated diseases [1]. Evidence indicates that the reduction of the levels of CoQ_10_ during aging can be associated with cardiovascular disease, type II diabetes, metabolic disease, sarcopenia, neurodegenerative diseases and other age-related diseases [2]. In this special issue some aspects of the importance of CoQ_10_ supplementation during aging, especially in fertilization, neurodegeneration and cardiovascular diseases, are highlighted.

One of the important problems in human aging affects reproduction. As Ben-Meir et al., reported in this special issue, one of the important changes in human behavior during last decades is the delay in the reproductive age [3]. The success of embryonic development in women older than 35 years decreases significantly. This effect can be due to a decrease in the quality of oocytes but also in cumulus granulosa cells that surround oocytes in the ovary follicle that also suffer mitochondrial dysfunction during aging. This mitochondrial dysfunction is accompanied by a decrease in the levels of proteins involved in CoQ_10_ synthesis both in mice and in women. Ben-Meir and colleagues demonstrate that supplementation with CoQ_10_ can improve the quality of both, oocytes and cumulus granulosa cells affecting mitochondrial metabolism, restoring the decrease in CoQ_10_ levels and improving maternal age in a mice model [3].

Another contribution in this special issue deepens the importance of CoQ_10_ in the maintenance of fertility after 35 years of age. The work shown by Giannubilo and collaborators indicates that supplementation with CoQ_10_ in women treated with in vitro fertilization–embryo transfer procedure improved follicular content and its oxidative status [4]. Supplementation with 200 mg/day of CoQ_10_ for 30 days ended in higher levels in follicular fluid and oocytes with lower oxidation rate indicating a lower oxidative stress. The two works about fertility shown in this special issue highlight the importance of CoQ_10_ levels in the maintenance of fertility capacity in mature women [3,4].

CoQ_10_ plays also an important role in the prevention of cardiovascular disease and in the repair of damages after myocardial infarction [5,6]. In this special issue, supplementation with CoQ_10_ improves the remodeling, oxidative damage and other markers of damage in left ventricles in patients suffering acute myocardial infarction [7]. This study reinforces the importance of supplementation with CoQ_10_ just after acute myocardial infarction in order to protect cells against further damage and to improve the evolution of the disease by accelerating reparation of cardiovascular tissue. If we add this evidence to the importance of CoQ_10_ in the prevention of atherosclerosis, by reducing the oxidation of low-density lipoproteins, CoQ_10_ [8,9] must be seriously considered as an essential supplement in the prevention of morbidity and mortality by cardiovascular diseases, one of the main diseases in aging.

One of the most known dysfunctions associated with aging is the decrease in neurological capacity. It is widely known that age is associated with the decrease in neurological activity, probably associated with the decrease in mitochondrial function. In this special issue, supplementation with a water-soluble CoQ_10_ derivative is shown as a promising anti-aging agent by improving age-related brain disorders such as cognitive decline or Alzheimer disease [10]. The main problem in CoQ_10_ supplementation is the use of formulations able to show high bioavailability and to increase CoQ_10_ levels in tissues suffering mitochondrial dysfunction during aging [11]. In this interesting revision, Takahashi and Takahashi show that the incorporation of CoQ_10_ in the central nervous system is important in the prevention of neurodegenerative diseases such as Alzheimer, Parkinson or encephalopathy. This review shows that improved formulations of CoQ_10_, able to increase its levels in the central nervous system, can be very effective in the prevention of neurological disorders during aging [10].

The importance of CoQ_10_ in longevity and age-relate degenerative disorders is highlighted by Mantle and Hargreaves [12]. In this review, authors show the main effects of CoQ_10_ found in elderly people. Apart from the effects already mentioned in fertilization and cardiovascular disease, this review also indicates the importance of CoQ_10_ supplementation in the treatment of glycaemic control and vascular function in type II diabetic patients. Further, supplementation with CoQ_10_ also improves renal function in chronic kidney disease, reduces liver inflammation in non-alcoholic liver disease and other life-style related diseases associated with mitochondrial dysfunction.

Interestingly, all these effects of CoQ_10_ are associated with mitochondrial dysfunction-related diseases, possibly aggravated by a reduction of CoQ_10_ levels by impaired synthesis during aging. Supplementation with this molecule by using high-bioavailability preparations would delay age-related progression and improve aging-associated physiological deterioration. In this special issue, only a few articles summarize some of the main aspects that can be improved with the supplementation with CoQ_10_. Further research is needed, but evidence about the importance of this molecule in several diseases, aging and age-related diseases demonstrates the importance of maintaining CoQ_10_ levels at old ages. 
